# The influence of training status on right ventricular morphology and segmental strain in elite pre-adolescent soccer players

**DOI:** 10.1007/s00421-021-04634-3

**Published:** 2021-02-22

**Authors:** Viswanath B. Unnithan, Alexander Beaumont, Thomas W. Rowland, Nicholas Sculthorpe, Keith George, Rachel Lord, David Oxborough

**Affiliations:** 1 Division of Sport and Exercise, Institute of Clinical Exercise and Health Science, School of Health and Life Sciences, University of the West of Scotland, Hamilton, Scotland, UK; 2 School of Sport, York St. John University, York, UK; 3 Research Institute for Sport and Exercise Sciences, Liverpool John Moores University, Liverpool, UK; 4 Cardiff Centre for Exercise and Health, Cardiff Metropolitan University, Cardiff, UK

**Keywords:** Youth soccer, Right ventricle, Longitudinal strain, Segmental analyses

## Abstract

Cardiac modifications to training are a product of the genetic pre-disposition for adaptation and the repetitive haemodynamic loads that are placed on the myocardium. Elite pre-adolescent athletes are exposed to high-intensity training at a young age with little understanding of the physiological and clinical consequences. It is unclear how right ventricular (RV) structure and function may respond to this type of stimulus. The aim of this study was to compare RV structure and strain across the cardiac cycle and within individual segments in elite soccer players (SP) and controls (CON). Methods: Twenty-two highly trained, male pre-adolescent SP and 22 age-and sex-matched recreationally active individuals CON were investigated using 2D echocardiography, including myocardial speckle tracking to assess basal, mid-wall, apical and global longitudinal strain and strain rate during systole (SRS) and diastole (SRE and SRA). Results: greater RV cavity size was identified in the SP compared to CON (RVD_1_ SP: 32.3 ± 3.1 vs. CON: 29.6 ± 2.8 (mm/m^2^)^0.5^; *p* = 0.005). No inter-group differences were noted for peak global RV strain (SP: − 28.6 ± 4.9 vs CON: − 30.3 ± 4.0%, *p* = 0.11). Lower mid-wall strain was demonstrated in the SP compared to CON (SP: − 27.9 ± 5.8 vs. CON: − 32.2 ± 4.4%, *p* = 0.007). Conclusion: Soccer training has the potential to increase RV size in pre-adolescent players. The unique segmental analyses used in this study have identified inter-group differences that were masked by global strain evaluations. The clinical and physiological implications of these findings warrant further investigation.

## Introduction

Pre-adolescent soccer players are exposed to high training demands at a young age, with reported intensities surpassing 85% maximal heart rate during intermittent high-intensity training and match-play (Wrigley et al. 2012). Furthermore, during elite age-group (12 years) match-play, total distances covered are approximately 5000 m of which, approximately one third (1700 m) are covered at high speed (Doncaster and Unnithan 2019). The combination of internal and external loads associated with elite level soccer at a young age place a significant physiological stress on the cardiovascular system.

Cardiac adaptation to exercise training is a product of the training exposure (mode, intensity, duration and volume) and the ensuing repetitive haemodynamic loads that are placed on the myocardium (Weiner and Baggish 2012). Most of the work in the pre-adolescent athlete has focussed on the left side of the heart, where evidence exists of both structural (Unnithan et al. 2018) and functional adaptations (Beaumont et al. 2019) in the elite pre-adolescent soccer player compared to matched controls. It is unclear what impact exercise training and maturation will have on right ventricular (RV) morphology and global and regional function. In addition, the relationship between RV morphology and function as determinants of soccer performance, such as aerobic capacity in the pre-adolescent soccer player (Doncaster and Unnithan 2019) is unknown.

Within the athletic pre-adolescent population, a paucity of data exists that characterises differences in RV morphology between trained and untrained populations. The limited research that does exist, has been restricted to the endurance athlete (D’Ascenzi et al. 2017), where a larger right ventricular outflow tract (RVOT) diameter has been observed, compared to recreationally active peers. This phenomenon was also noted in older, adolescent (16 years) SP compared to their age-matched controls (Popple et al. 2018), suggesting that such morphological adaptations appear to be driven by training-induced changes rather than any maturational threshold.

Current evidence of differences in global and regional RV function in the pre- adolescent athlete have been demonstrated by lower RV fractional area change (RVFAC) in endurance trained (swimmers) compared to untrained pre-adolescents (D’Ascenzi et al. 2017), with no inter-group differences in peak global longitudinal strain (*ε*). The use of peak RV strain, both globally and segmentally, has recently been used in clinical adolescent populations and is potentially a useful diagnostic tool (Pieles et al. 2019).

While identification of peak ε values provides some insight of global function, ε analysis applied across the entire cardiac cycle allows for a temporal assessment of RV *ε*, which facilitates a more nuanced insight into RV ventricular mechanics than that seen by peak values alone (Utomi et al. 2014; Johnson et al. 2018). We have previously reported that temporal analysis of ε and strain rate (SR) in the LV discriminated between SP and controls in sections of the cardiac cycle where peak values were unable to (Beaumont et al. 2019). Moreover, existing work in this area has averaged all myocardial segments in the assessment of cardiac ε (D’Ascenzi et al. 2017). A meta-analysis, however, in healthy children ε reported that ε is heterogenous in the RV, demonstrating a base-to-apex gradient (highest ε at the base and lowest at the apex) (Levy et al. 2014). Whether segment specific adaptations in the athletic RV free wall (FW) are present are not known and warrant detailed investigation.

Evaluating the relationship between aerobic capacity, morphology and function of the RV is important, as aerobic capacity has previously been identified as a significant predictor of soccer performance in pre-adolescent SP (Doncaster and Unnithan 2019). Indeed, research has demonstrated significant, but weak correlations between RV morphological markers, global RV function and aerobic capacity in adult team sport players (20 years) (Lazic et al. 2019). This question has not been explored using speckle tracking echocardiographic (STE) markers of RV ε and SR as determinants of aerobic capacity in pre-adolescent soccer players.

The primary aim of the study was to compare RV morphology, global and regional RV function and peak and temporal ε between a group of elite pre-adolescent SP and a group of recreationally active, but not systematically trained, age-matched control participants (CON). The secondary aim was to explore the relationship between these RVindices and a surrogate measure of physical soccer performance (aerobic capacity). It was hypothesised that: (1) RV dimensions would be greater in the SP compared to the CON (2) global and temporal RV function would not be different when comparing the two groups and; (3) there would be significant relationships between RV morphology and function and aerobic capacity respectively.

## Material and methods

### Participants

Twenty-two highly trained, male youth SP and 22 age- and sex-matched recreationally active individuals (CON) were recruited to participate in the study. The SP were recruited from two Category one Premier League youth soccer academies. Category one academies represent the highest achievable grade for professional youth soccer academies in the English Premier League. The CON participants were recruited from a local high school close to one of the Premier League Academy training grounds.

The SP training profiles were as follows: 4.5 ± 1.5 years training, 11 ± 1 months per year training, 4 ± 1 training sessions per week and 9.4 ± 2.4 h per week of training. This volume of exercise training had been consistent for the entirety of their active training years. SP played one competitive match per week and had been engaged in competitive soccer matches for 4 ± 2 years. For one club, 14 boys from the Under-12 (U12) squad and their parents were approached, of which three were not enrolled because of either personal circumstance (*n* = 2) or a soccer related injury (*n* = 1). At the second club, researchers provided information to 15 U12 players and their parents, of which 2 were recovering from injury, one was released from the club after signing up from the study, and one signed up and simply did not attend the testing. This resulted in 11 participants from both clubs, with a total of 22 SP volunteering for the study. CON participants took part in compulsory physical education of 2 h per week (the same as SP), were all recreationally active and without engagement in systematic training. The CON self-reported 1.53 ± 1.77 h per week of physical activity (Beaumont et al. 2019).

All participants underwent a physical examination and completed a medical history questionnaire. Exclusion criteria included the use of any medications that would influence cardiovascular function and any personal or early family history of cardiovascular disease. Written informed parental and participant consent was obtained prior to participation. All procedures performed in the study were in accordance with the ethical standards of the Declaration of Helsinki and the study was reviewed and approved by a local University ethics committee.

### Study design

The study employed a prospective, cross-sectional, cohort assessment of cardiac structure and function in highly trained pre-adolescent SP and CON. All testing took place at the training grounds of the two soccer clubs and at a local school for the CON participants. Participants were instructed to refrain from exercise on the day preceding the test. Furthermore, all participants were also informed to refrain from consuming any drinks containing sugar or caffeine, as well as the consumption of any food in the two hours preceding the testing session.

### Protocol/measurements

Physical activity and training questionnaires (Rowland et al. 2009) were completed prior to the testing. Following this, stature, sitting height and body mass were measured. Maturity status was quantified using maturity offset (Sherar et al. 2005; Unnithan et al. 2018). Resting arterial blood pressure was recorded in the left arm by an automated blood pressure cuff (Boso, Medicus, Jungingen, Germany) and heart rate was assessed by a 12-lead electrocardiogram (ECG) (CardioExpress SL6, Spacelabs Healthcare, Washington US). Resting echocardiographic measurements were taken in the left lateral decubitus position. All SP (*n* = 22) and a sub-sample (*n* = 15) of the CON participants underwent an assessment of peak oxygen uptake ($\dot{\mathrm{V}}{\mathrm{O}}_{2\mathrm{peak}}$) to reflect maximal aerobic capacity (Cortex MetaMax 3B, Cortex Biophysik GmbH, Leipzig, Germany) on a cycle ergometer (Lode, Corival, Groningen, Netherlands) (Unnithan et al. 2018).

#### Transthoracic echocardiography

All echocardiographic examinations were performed with the subject in the left lateral decubitas position using a commercially available ultrasound system (Vivid-Q, GE Healthcare, Horton, Norway) by experienced sonographers (DO, RL), to maximise quality control. A complete echocardiographic study was performed with an additional focus of the right-sided heart and all images were acquired and analysed in accordance with the American Society of Echocardiography (ASE) (Rudski et al. 2010; Lang et al. 2015). Images were stored in a raw Digital Imaging and Communications in Medicine (DICOM) format and exported to an offline analysis system (EchoPac, GE Healthcare, Horton, Norway, Version 202). Subsequent data analysis was performed by DO and AB using an average of 3 cardiac cycles for all conventional structure and function measures and one cardiac cycle for all strain measurements.

#### Standard conventional 2D doppler and tissue doppler imaging

**Fig. 1:**
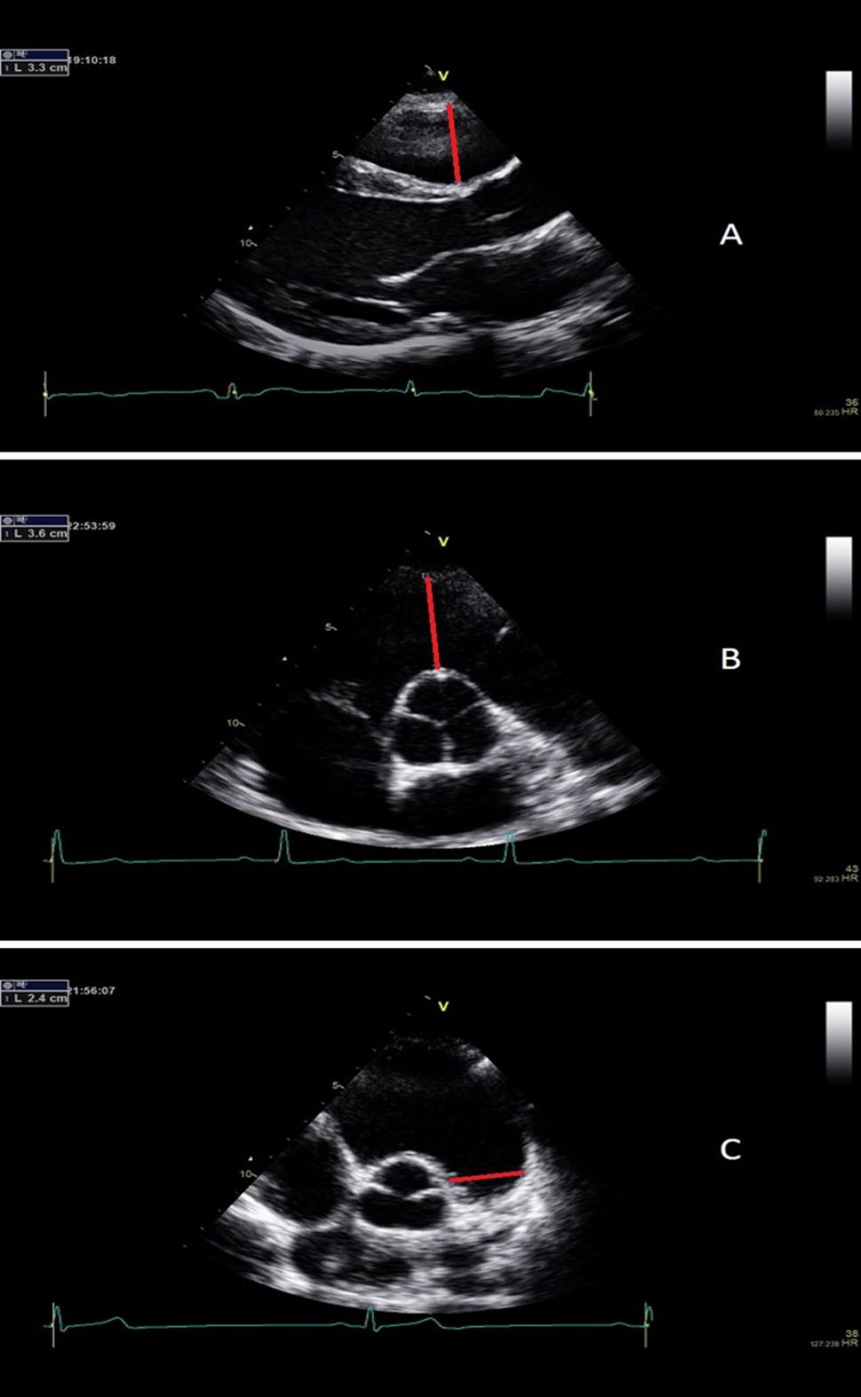
Parasternal views demonstrating RV outflow dimensions. **a** (Proximal) = RVOTplax; (**b**) (Proximal) = RVOT1; (**c**) (Distal) = RVOT2, Exemplar dimensions

**Fig. 2:**
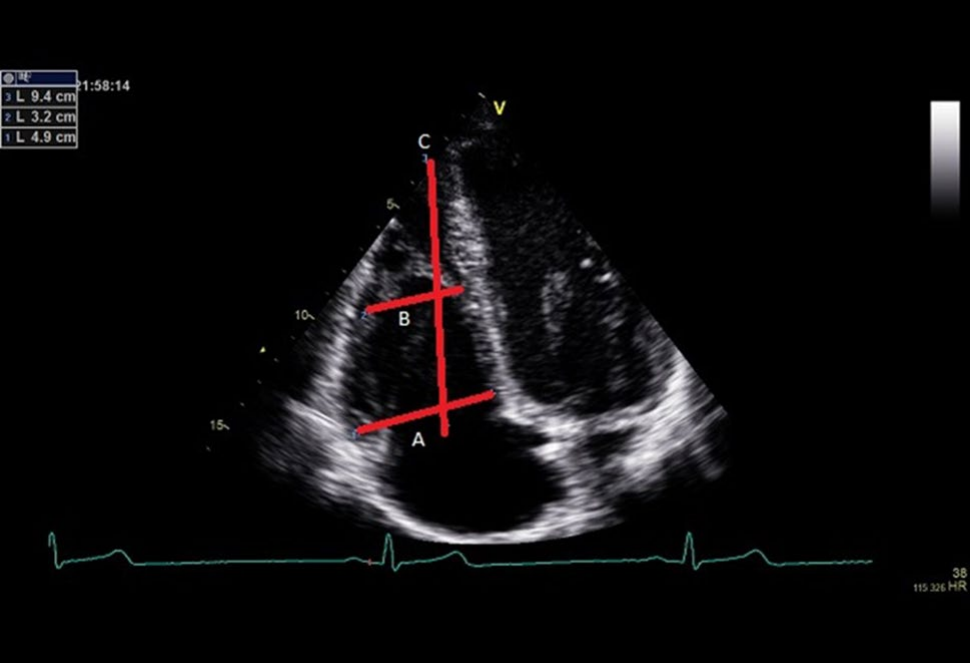
RVD1 was obtained at the level of the tricuspid annulus, RVD2 at the level of the LV papillary muscles and RD3 was measured from the apex to the tricuspid annulus **a** = RVD1; **b** = RVD2; **c** = RVD3, Exemplar dimensions

The parasternal long and short axis orientations were used to establish RV outflow tract dimensions (Fig. 1) at the proximal level from a parasternal long axis (RVOT_plax)_ and parasternal short axis (RVOT_1_), as well as distal level (RVOT_2_). A modified apical four chamber orientation was used to obtain measurements from the main body of the RV (Fig. 2) and included dimensions at the RV base (RVD_1_), mid cavity (RVD_2_) and RV length (RVD_3_). In addition, RV area was calculated in diastole (RVD_area_) and systole (RVS_area_) allowing the calculation of fractional area change (RVFAC). A 4 mm pulsed wave Doppler sample volume was placed at the level of the sub-pulmonary valve in the RV outflow tract allowing the assessment of the velocity time interval (RVOT_VTI_) and identification of pulmonary value closure (PVC), as the timing selected when the ascending side of pulmonary valve outflow trace reached baseline. Pulsed wave TDI was used to interrogate the RV lateral wall with a 2 mm sample volume positioned within the tricuspid annulus. Peak systolic (TDI-S’adj), early diastolic (TDI-E’adj) and late diastolic (TDI-A’adj) myocardial velocities were measured. RV: LV ratio was determined from basal measurements in the RV and LV taken from an apical 4 chamber view at end diastole (Popple et al. 2018). All chamber sizes were allometrically scaled to control for inter-individual differences in body surface area, according to the principles of geometric similarity (Batterham et al. 1999).

#### Myocardial speckle tracking

A modified apical four chamber with RV focus was utilised for assessment of ε and SR in the RV. Images were optimised using depth, gain, compression and sector width to provide optimal endocardial delineation. The focal point was positioned mid cavity to reduce the impact of beam divergence and frame rates were set between 80 and 90 frames per second. Offline analysis involved placing a region of interest along the RV lateral wall (FW) from base-to-apex. The software automatically tracked 3 RV FW segments (base, mid and apex) and provided an interpretation on tracking quality. Furthermore, the operator provided a subjective assessment of tracking quality and segments were excluded if deemed unacceptable. The peak values for *ε* and SR during ventricular systole (SRS) and early (SRE) and late ventricular diastole (SRA) were reported for the three wall segments and averaged to obtain a global value (Lang et al. 2015). Intra-observer coefficients of variation of between 7 and 12% have previously been established for RV strain by the research team (Lord et al. 2014; Oxborough et al. 2012a).

Following speckle tracking analysis, the resulting raw *ε* and SR files were imported into custom software (RV-SAS, Strain Analysis Solutions. Glasgow, UK). This software uses the speckle tracking data generated by Echopac and automates the analysis of the right ventricular changes. For each cardiac cycle, the software extracted ε data for each of the three segments of the RV FW only. Thus, the software also averaged ε values at each time point for each of the 3 FW segments (basal, mid-wall and apical) to provide an averaged value of ε and SR. For each of the 4 sets of data (individual 3 segments plus the averaged RV FW) the software applied a 500-point cubic spline to both the systolic and diastolic periods of the cardiac cycle (derived from PVC). The splined data was then used to identify systolic and diastolic peaks and relative timings for each of the 4 data sets. To facilitate temporal analysis, the software also calculated ε and SR values at 5% increments of the systolic and diastolic phases, respectively. Internal assessment of the software in our lab has found extremely strong agreement with peak ε and time to peak ε taken directly from Echopac’s analysis screens (r > 0.98, CoV < 1%).

### Statistical analysis

Normality of data was assessed using the Shapiro–Wilk test. For normally distributed data, a Student’s independent *t* test was used to compare RV structure, function, *ε* and SR in SP and CON. TDI-A’ was not normally distributed and consequently a Mann Whitney *U* test was performed to evaluate inter-group differences. The relationship between RV morphology and function (ε and SR) and their relationship with $\dot{\mathrm{V}}{\mathrm{O}}_{2\mathrm{peak}}$ was determined with Pearson correlation coefficients. A sample size of 22 SP provided a (1–β) of 80% at an alpha level of 0.05. Cohen’s *D* effect sizes were calculated for all inter-group comparisons. All statistical data was analysed using SPSS version 23.0, Chicago IL), with statistical significance granted at *p* < 0.05.

## Results

There was no inter-group difference for body mass (SP: 40.2 ± 5.8 kg vs. CON: 44.0 ± 11.7 kg) and stature (SP: 151.3 ± 6.3 cm vs. CON: 148.6 ± 7.3 cm). Body surface area (SP: 1.29 ± 0.12 m^2^ vs. CON: 1.33 ± 0.18 m^2^) was lower (*p* = 0.026) in the SP compared to CON. Resting heart rate was lower (*p* = 0.004) in the SP than CON (65 ± 8 beats.min^−1^ vs. 74 ± 10 beats.min^−1^, respectively). There was no significant inter-group difference in resting systolic (SP: 100 ± 8 vs. CON: 105 ± 13 mmHg) or diastolic (SP: 61 ± 9 vs. CON: 61 ± 10 mmHg) blood pressure. The SP demonstrated a higher (*p* = 0.0006) $\dot{\mathrm{V}}{\mathrm{O}}_{2\mathrm{peak}}$ than CON (48.0 ± 5.0 vs. 40.1 ± 7.5 mL·kg^−1^ ·min^−1^, respectively) although there was no inter-group difference for maximal HR (SP: 189 ± 7 vs. CON: 186 ± 9 beats.min^−1^).

### Right ventricular structure and function

Larger RVD_1_, RVD_2_ and RVD_3_ were noted in the SP compared to the CON in both absolute terms and after scaling (Table 1). The RV:LV ratio was greater (*p* = 0.001) in the SP compared to the CON (Table 1). RVFAC was greater (*p* = 0.02) in the SP compared to the CON (Table 1). There were no significant differences with regard to peak tissue velocities adjusted for RV length [TDI-S’adj: (SP) 0.17 ± 0.02 vs. (CON) 0.17 ± 0.04 cm.s^−1^.mm^−1^], [TDI-E’adj: (SP) 0.21 ± 0.04 vs. (CON) 0.19 ± 0.04 cm.s^−1^.mm^−1^] and [TDI-A’adj: (SP) 0.11 ± 0.03 vs (CON) 0.11 ± 0.01 cm.s^−1^.mm^−1^].

**Table 1:** Right ventricular structure and function

	SP (*n* = 22)	CON (*n* = 22)	*p* value	ES (Cohen’s D)
RVOTplax (mm)	24.0 ± 2.8	23.8 ± 2.8	0.661^a^	0.08
RVOT Plax index (mm/m^2^)^0.5^	21.2 ± 2.6	20.6 ± 1.9	0.195^a^	0.25
RVOT_1_ (mm)	26.5 ± 3.4	26.3 ± 2.5	0.801	0.08
RVOT_1_ index (mm/m^2^)^0.5^	23.4 ± 3.0	22.7 ± 1.9	0.419	0.25
RVOT_2_ (mm)	20.8 ± 3.1	19.9 ± 2.2	0.401^a^	0.34
RVOT_2_ index (mm/m^2^)^0.5^	18.4 ± 2.9	17.3 ± 1.7	0.333^a^	0.46
RVD_1_ (mm)	36.6 ± 3.7	34.1 ± 3.4	**0.025**	0.70
RVD_1_ index (mm/m^2^)^0.5^	32.3 ± 3.1	29.6 ± 2.8	**0.005**	0.90
RVD_2_ (mm)	27.5 ± 3.0	25.3 ± 3.0	**0.020**	0.73
RVD_2_ index (mm/m^2^)^0.5^	24.2 ± 2.8	21.9 ± 2.5	**0.006**	0.88
RVD_3_ (mm)	76.6 ± 6.8	71.1 ± 7.5	**0.015**	0.76
RVD_3_ index (mm/m^2^)^0.5^	67.5 ± 5.2	61.7 ± 5.8	**0.001**	1.06
RVDarea (cm^2^)	17.5 ± 2.6	17.1 ± 3.6	0.634	0.14
RVDarea index (cm^2^/m^2^)^1.0^	13.6 ± 1.7	12.8 ± 2.2	0.195	0.40
RVSarea (cm^2^)	9.0 ± 1.5	9.6 ± 2.3	0.510^a^	0.30
RVFAC (%)	49 ± 6	44 ± 7	**0.020**	0.73
RV:LV	0.92 ± 0.08	0.81 ± 0.09	**0.001**	1.25

Bold values denote *p*<0.05

Data are mean ± standard deviation

All index values allometrically scaled to body surface area

*RVOTplax* RV outflow tract dimensions at the proximal level from a parasternal long axis view; *RVOT*_*1*_ RV outflow tract dimensions parasternal short axis view; *RVOT*_*2*_* RV* outflow tract dimensions at the distal level. *RVD*_*1*_ RV base, (RVD_2_) mid cavity and *RVD*_*3*_ RV length. *RVD*_*area*_ RV area in diastole and *RVS*_*area*_ systole. (RV_FAC_) fractional area change. *RV:LV* ratio

^a^Not normally distributed data and the application of the Mann–Whitney test

### Peak global RV and segmental strain and strain rate

**Fig. 3:**
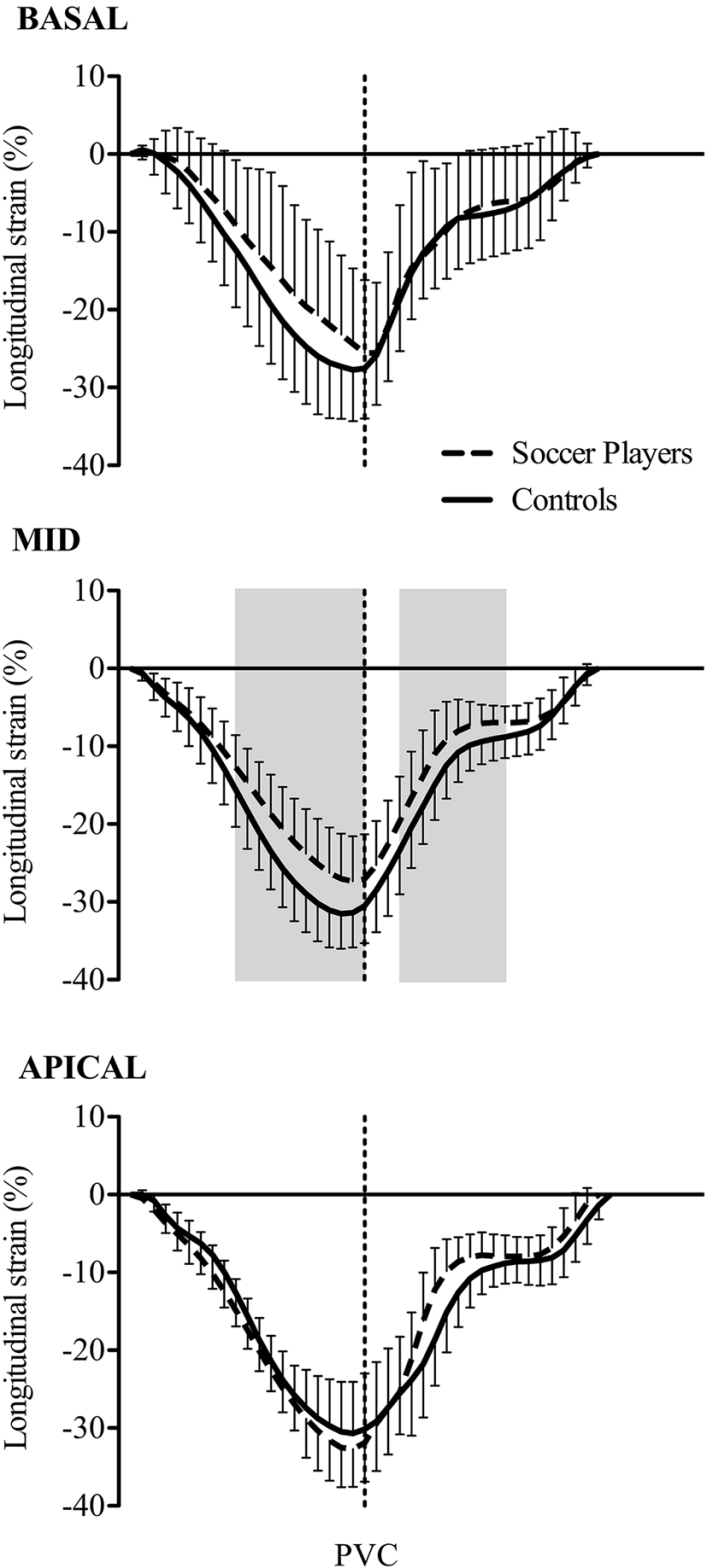
Temporal analyses across the cardiac cycle. Shaded areas in MID demonstrate statistically significantly (*p* < 0.05) lower mid-wall longitudinal strain from Systole 50–95% and Diastole 10–60% in the SP (– , –, –) compared to CON (−)

There was no inter-group difference in peak global longitudinal ε. There was a longer (*p* = 0.04) time to peak for global longitudinal ε in the SP compared to CON (Table 2). Lower global SRS (*p* = 0.00007) and SRA (*p* = 0.005) respectively were also identified in the SP compared to CON (Table 2). Lower (*p* = 0.007) MW peak longitudinal ε was identified in the SP compared to the CON. Lower peak MW SRS (*p* = 0.0014) and MW SRA (*p* = 0.0039) were noted in SP compared to CON. Apical time to peak *ε* was longer (*p* = 0.02) in the SP compared to CON. Apical SRE was (*p* = 0.04) greater in SP compared to CON (Table 2). Temporal analyses across the cardiac cycle (Fig. 3) demonstrated lower (*p* < 0.05) mid-wall ε (see shaded areas in MID- Fig. 3) from Systole 50–95% and Diastole 10–60% in the SP compared to CON. Greater ε was noted at the apex in the SP (Fig. 3. Apical) and these approached significance at 35% diastole (*p* = 0.0628) and 40% diastole (*p* = 0.0656).

**Table 2:** Peak RV and segmental strain and strain rates

	SP (*n* = 19)	CON (*n* = 19)	*P* value	ES (Cohen’s D)
Peak Global RV ε and strain rate				
RV *ε* (%)	− 28.6 ± 4.9	− 30.3 ± 4.0	0.11	0.377
Time to Peak (s)	0.36 ± 0.02	0.35 ± 0.03	**0.04**	0.39
RV SRS (l/s)	− 1.6 ± 0.3	− 2.1 ± 0.4	**0.00007**	1.41
RV SRE (l/s)	2.0 ± 0.5	2.1 ± 0.7	0.60	0.16
RV SRA (1/s)	0.8 ± 0.5	1.2 ± 0.5	**0.005**	0.80
Peak segmental strain and strain rate				
Basal *ε* (%)	− 27.2 ± 7.6	− 28.4 ± 7.1	0.36	0.164
Basal Time to Peak (s)	0.37 ± 0.08	0.35 ± 0.03	0.55	0.331
Basal SRS (1/s)	− 2.0 ± 0.3	− 2.1 ± 0.5	0.29	0.242
Basal SRE (1/s)	2.7 ± 0.7	2.9 ± 0.8	0.51	0.266
Basal SRA (1/s)	1.3 ± 0.6	1.4 ± 0.9	0.46	0.131
Mid-Wall (MW)				
MW ε (%)	− 27.9 ± 5.8	− 32.2 ± 4.4	**0.007**	0.835
MW Time to Peak (s)	0.36 ± 0.02	0.35 ± 0.03	0.26	0.392
MW SRS (1/s)	− 1.6 ± 0.4	− 2.1 ± 0.5	**0.00014**	1.10
MW SRE (1/s)	2.1 ± 0.8	2.2 ± 0.5	0.59	0.150
MW SRA (1/s)	0.9 ± 0.4	1.2 ± 0.4	**0.0039**	0.750
Apical				
Apical ε (%)	− 33.4 ± 5.1	− 31.0 ± 6.3	0.34	0.418
Apical Time to Peak (s)	0.35 ± 0.02	0.33 ± 0.03	**0.02**	0.784
Apical SRS (1/s)	− 2.1 ± 0.6	− 2.4 ± 0.6	**0.05**	0.500
Apical SRE (1/s)	2.6 ± 0.7	2.2 ± 1.0	**0.014**	0.463
Apical SRA (1/s)	1.2 ± 0.6	1.4 ± 0.6	0.32	0.333

Bold values denote *p*<0.05

Data are mean ± SD

(ε) Peak longitudinal strain and (*SR*) strain rate. (*SRS*) strain rate during systole and (SRE) during early diastole and late diastole (*SRA*)

#### Peak global and segmental strain and strain rate relationships with aerobic capacity and heart rate

Moderate, but significant correlations were derived between $\dot{\mathrm{V}}{\mathrm{O}}_{2\mathrm{peak}}$ and peak global SRS (*r* = 0.519, *p* = 0.001), peak MW SRS (*r* = 0.503, *p* = 0.002) and apical SRS (*r* = 0.433, *p* = 0.015), respectively. There were no other significant correlations between $\dot{\mathrm{V}}{\mathrm{O}}_{2\mathrm{peak}}$ and RV morphology, RV ε and SR. Significant correlations were obtained between peak global longitudinal ε and resting heart rate (*r* =  − 0.479, *p* = 0.000) and MW *ε* and resting heart rate (r =  − 0.553, *p* = 0.000).

## Discussion

The major findings from this study were: (1) larger RV dimensions in the SP compared to CON. (2) higher RVFAC in the SP compared to CON. (3) no inter-group difference for global peak longitudinal *ε*, however, interrogation of the RV using segmental ε analyses demonstrated lower peak ε in the SP compared to CON in the MW region of the RV. (4) lower MW *ε* was noted in mid-to-late systole and early diastole in the SP compared to CON and (5) significant correlations between $\dot{\mathrm{V}}{\mathrm{O}}_{2\mathrm{peak}}$ and global and segmental SR.

### RV structure

The morphological enlargements in RV cavity diameter noted in the SP in the present study are likely driven by repeated exposure to elevations in RV end-systolic wall stress from relative increases in pulmonary artery pressures (La Gerche et al. 2011). Additionally, persistently increased RV pre-load may also have contributed to the larger RV cavity dimensions observed in SP than CON. Previous evidence does exist with respect to morphological adaptations in pre-adolescent athletes, but this was noted in pre-adolescent swimmers (D’Ascenzi et al. 2017), where the horizontal body position of the sport would accentuate an increased pre-load and drive RV adaptation. In contrast to the present study, after allometric adjustments were made for body surface area, the observed RV enlargements in swimmers compared with controls were less obvious. The presence of morphological adaptations in the present study occurred in individuals exposed to soccer training (upright body position) and this would suggest that the intensity and volume of the training rather than any orthostatic stimulus could be the factor driving RV adaptation in these players (Qasem et al. 2018).

In the present study, the lack of difference in outflow tract dimensions coupled with a bigger basal inflow (RVD_1_) dimensions in SP compared to CON mimics the pattern seen in adult endurance athletes (Oxborough et al. 2012b). This concurs with the type of adaptation anticipated from the training modes commonly used in repeat sprint type sports where there is a greater dynamic than static component in training and match-play (Utomi et al. 2015). Longitudinal data measured over the course of an 8-month competitive season in adult volleyball and basketball players demonstrated differential RV adaptation during the training season (D’Ascenzi et al. 2016) whereRVD_1_ and RVD_2_ dimensions increased, but RVOT did not. Taken together, these longitudinal data support cross-sectional data generated in the present study that RV inflow and mid cavity are more sensitive to a high-intensity training stimulus than RVOT. In line with the present investigation, unremarkable differences in RVOT consequent to a chronic pre-adolescent training stimulus in swimmers were noted with matched CON (D’Ascenzi et al. 2017).

Unequal remodelling (RV:LV) has previously been seen in adult endurance athletes (La Gerche et al. 2011; Oxborough et al. 2012b) (Arbab-Zadeh et al. 2014). In the present study, RV enlargement was disproportionate to that of the LV (Unnithan et al. 2018) in the SP compared to CON, based on the greater RV:LV ratio seen in SP. The increased RV: LV ratio in SP in conjunction with the recently reported unremarkable differences in LV chamber diameter between SP and CON (Beaumont et al. 2019) in the same cohort of SP and CON suggests that the major adaptation to soccer training would appear to occur in the RV rather than the LV. The bases for these changes may be a product of an enhanced pre-load and disproportionate wall stress-induced adaptations in the RV.

### RV function

The present study is one of the first to explore functional differences with respect to training status in this population (D’Ascenzi et al. 2017). Conventional indices of global RV function such as RVFAC were larger in SP compared to CON. These findings oppose evidence in pre-adolescent swimmers that demonstrated no training-induced increases in RVFAC in response to 5-months of intensive swimming training, and lower RVFAC in the swimmers at baseline compared to age-matched controls (D’Ascenzi et al. 2017). It is possible to speculate that in the present study, the combination of a low HR at rest and/or RV enlargement in the SP required an increase in RV contractility to maintain an adequate resting cardiac output (Frank-Starling Law).

The lack of difference in peak global longitudinal strain between SP and CON is in accordance with previous findings in competitive-level pre-adolescent swimmers that demonstrated no differences vs age-matched control participants prior to the onset of training (D’Ascenzi et al. 2017). Similarly, evidence exists in adults of either reduced (La Gerche et al. 2012; King et al. 2013) or no difference in RV strain (Oxborough et al. 2012b; Utomi et al. 2015) when comparing athletic and non-athletic individuals. In concert, the lack of inter-group differences in ε and the superior RVFAC seen in the SP suggest that a direct measure of global longitudinal function (peak global longitudinal ε) is unaffected by physiological adaptation and further suggests a possible greater impact on circumferential shortening. Furthermore, RVFAC (similar to LV ejection fraction) is more dependent on ventricular load and chamber size and therefore our findings may also, in part, reflect this.

The present study extends existing work (D’Ascenzi et al. 2017) by incorporating segmental and temporal analysis of the RV FW in an adolescent athletic population. We observed that SP demonstrated lower MW shortening ε than CON, yet ε in basal and apical segments were unremarkable between groups. In comparison with existing work that assessed segmental RV strain in healthy adolescents (13 years) (Pieles et al. 2019) our values are lower at basal, MW and apex. These differences could be due to the vendor-related differences in 2D strain- derived indices (Nagata et al. 2015).

Such differences in mid-wall *ε* between SP and CON are evident not just at peak but throughout the latter half of systole. These data highlight an important and previously undocumented advancement in our current understanding of RV ε in the athletic heart, by evidencing that sole reporting of peak global mechanics may conceal region specific adaption. The physiological significance of these differences and similarities between trained and untrained hearts of pre-adolescents requires further investigation, yet could represent a contractile reserve important during exercise, and mimics a pattern seen within basal and mid-wall segments in adult endurance athletes (Teske et al. 2009). Peak global longitudinal ε was inversely associated with resting HR. This relationship was influenced by the significant inverse relationship noted between MW strain and HR, thereby creating more supportive evidence for a possible contractile reserve for the RV during exercise (La Gerche et al. 2011).

Similarly, interrogation of the SR segmental analyses identified the potential mechanism for globally reduced SRS in SP than CON. It would appear that the slower peak RV SRS was a product of a slower MW peak SRS in the SP, without differences in other RV FW segments between groups. Similar segmental SR findings were noted in the mid-wall segment in adult endurance athletes (Teske et al. 2009). It is possible to speculate that as the larger RVD_2_ of the SP has a greater number of myofibrils, any given level of deformation will take a longer time period; hence a slower SR will result (Maciver and Townsend 2008). Consequently, wall tension and intraventricular pressure during systole will be generated or released at a slower speed. During diastole, however, segmental analyses allowed the further interrogation into the source of the slower peak RV SRA in the SP, which was principally determined by slower mid-wall SRA. This apparently greater late diastolic function in SP may be the consequence of superior SRE during the early phases of ventricular relaxation, specific to the apex. Indeed, temporal analysis of strain revealed that SP showed a trend (*p* = 0.06) towards greater lengthening at 35% diastole. This may be coupled with the enhanced SRE in SP than CON, driving the early diastolic pressure gradient between RA and RV.

### Relationship between RV mechanics and aerobic capacity

The evidence of a significant relationship between $\dot{\mathrm{V}}{\mathrm{O}}_{2\mathrm{peak}}$ and global RV ε observed in the present study has also been demonstrated in the LV in healthy adolescents (Pieles et al. 2015) and elite soccer referees (Gianturco et al. 2017). There is only one study to date that demonstrated weak, but significant relationships between global RV function and $\dot{\mathrm{V}}{\mathrm{O}}_{2\mathrm{peak}}$ in adult team sport players (Lazic et al. 2019). There is no concomitant data in the RV for elite pre-adolescent athletes. Furthermore, significant positive relationships in the present study were also identified between $\dot{\mathrm{V}}{\mathrm{O}}_{2\mathrm{peak}}$ and MW and apical SRS, respectively at rest. MW and apical SRS at rest were also both lower in the SP compared to CON. The SRS data generated in the present study were obtained at rest. There is, however, evidence that RV SRS increases progressively in proportion to the increase in pulmonary artery pressures during exercise (La Gerche et al. 2012). The combined evidence from the cross-sectional analyses between the SP and CON *ε* (no inter-group differences or lower in SP) and SRS global and segmental findings (lower in SP compared to CON), their subsequent relationships with $\dot{\mathrm{V}}{\mathrm{O}}_{2\mathrm{peak}}$ (moderate positive relationships) and evidence from the extant literature (La Gerche et al. 2012), suggests that a contractile reserve may exist in the SP. This functional reserve may allow the SP to generate the intrinsic RV contractility during exercise required for the high aerobic capacity needed in elite youth soccer.

### Limitations and implications for future research

As with all cross-sectional studies, the morphological and functional differences identified in this study ascribed potentially to high-intensity soccer training, could also be a product of genetic self-selection. Consequently, there is a need for a longitudinal evaluation of the effect of high-intensity soccer training on changes in RV morphology and function in males and females, as well as athletes of different ethnicities. Furthermore, the use of the cycle ergometer to obtain the $\dot{\mathrm{V}}{\mathrm{O}}_{2\mathrm{peak}}$ data in the current study may have slightly under-estimated the aerobic capacity and associated cardio-dynamic changes in the SP. This modality was selected as it allowed us to capture in-exercise left ventricular ε and Tissue-Doppler imaging without excessive upper body motion in the same cohort (Unnithan et al. 2018). The present study identified lower global SRS in the SP compared to CON, interrogation of ECG data will allow future studies to provide possible mechanistic insight into these differences. The lack of inter-group differences in global peak ε suggests that a direct measure of global longitudinal function is unaffected by physiological adaptation. In conjunction with the superior RVFAC seen in the SP, this may suggest that high-intensity training has a possible greater impact on circumferential rather than longitudinal shortening. Two dimensional echocardiography used in the present study is unable to determine circumferential strain. Consequently, future studies interrogating the circumferential strain response to high-intensity training in this population are warranted. The present study presents initial data that suggests this approach (global and segmental analyses) may facilitate a greater understanding of the normative values of recreationally and highly fit pre-adolescents. Our findings highlighted an increased RV size and altered function in trained male pre-adolescent soccer players. Essentially these data are important when considering normal variance in this population. Echocardiography is used as a front-line investigation in the assessment of cardiomyopathy. Therefore, when considering RV structure and function to exclude conditions such as arrhythmogenic right ventricular cardiomyopathy (ARVC), it is pertinent to take into account athletic status. Our findings of increased RV size may make the structural differentiation challenging, but significantly, RV function was not depressed as is defined in the functional criteria for ARVC.

## Conclusions

Despite exposure to limited years of soccer training and a level of biological immaturity, this cross-sectional study demonstrated that pre-adolescent SP have larger RV dimensions than age- and sex-matched recreationally active peers. Furthermore, the novel segmental and temporal analyses adopted in this study demonstrated the first evidence of lower MW ε and SRS in SP compared to CON in this age group, despite unremarkable differences in global peak ε and SR between groups. This segmental and temporal analyses identified differences between trained and untrained groups, which would have been concealed with sole reporting of peak global longitudinal *ε*. The use of global and segmental strain analysis may help to define the normative values in pre-adolescents who are highly trained athletes.

## Data Availability

All participant data was anonymised using a custom coding system.

## Abbreviations

CON: Controls
ε: Peak longitudinal strain
FW: Free wall
LV: Left ventricle
MW: Mid wall
PVC: Pulmonary valve closure
RV: Right ventricle
RVD_area_: Right ventricular area calculated in diastole
RVD_1_: Right ventricular base
RVD_2_: Right ventricular mid cavity
RVD_3_: Right ventricular length
RVFAC: Right ventricular fractional area change
RVOT: Right ventricular outflow tract
RVOT_plax_: Right ventricular outflow tract parasternal long axis
RVOT_VTI_: Right ventricular outflow tract velocity–time integral
RVOT_1_: Right ventricular outflow tract short axis
RVOT_2_: Right ventricular outflow distal level
RVS_area_: Right ventricular area calculated in systole
SP: Soccer players
SR: Strain rate
SRA: Strain rate during late diastole
SRE: Strain rate during early diastole
SRS: Strain rate during systole
STE: Speckle tracking echocardiography
TDI: A’adj-Peak late diastolic myocardial velocity adjusted for RV length
TDI: E’adj-Peak early diastolic myocardial velocity adjusted for RV length
TDI: S’adj-Peak systolic myocardial velocity adjusted for RV length
${\dot{\text{V}}\text{O}}_{{2{\text{peak}}}}$: Peak oxygen uptake

## Availability of data and material

All raw data is available to be viewed in electronic and hard copy format.
